# Associations between teachers’ professional competencies and the quality of interactions and relationships in preschool: findings from Austria

**DOI:** 10.3389/fpsyg.2023.1222369

**Published:** 2023-11-23

**Authors:** Eva-Maria Embacher, Wilfried Smidt

**Affiliations:** Department of Psychosocial Intervention and Communication Studies, University of Innsbruck, Innsbruck, Austria

**Keywords:** interaction quality, relationship quality, professional competencies, beliefs, work engagement, self-efficacy, ECEC, preschool

## Abstract

The professionalization of preschool teachers is considered an important factor for ensuring and improving the quality of interactions and relationships. Findings on associations between teachers’ professional competencies and the quality of interactions and relationships in preschools are not only inconsistent in general but also rare for early childhood education and care (ECEC) in Austria. Therefore, the aim of this study is to address this research gap by considering interaction quality at the child level (measured with the inCLASS) and preschool teachers’ perceptions of the teacher–child relationship (measured with the STRS). A sample of 287 children from 89 Austrian preschools was examined. After including control variables, the results of regression analyses revealed that preschool teachers’ beliefs on co-construction were negatively related to task orientation, whereas their beliefs on instruction were positively related to task orientation. Furthermore, preschool teachers’ work engagement was positively related to conflict interactions. Regarding teacher–child closeness, a positive association with preschool teachers’ work engagement was found. Results on teacher–child conflict showed a positive effect of preschool teachers’ beliefs on instruction and negative effects of teachers’ beliefs on co-construction and their self-efficacy. The findings are discussed in regard to the professionalization of preschool teachers.

## Introduction

The quality of interactional processes that children experience with preschool teachers and peers in preschool^1^ is often termed “process quality” or “interaction quality” (Schmidt et al., 2018), and research has shown that it predicts children’s socioemotional, cognitive, and language-related competencies [Tietze et al., 1998; National Institute of Child Health and Human Development, and Early Child Care Research Network (NICHD ECCRN), 2006; Sylva et al., 2006; Burger, 2010; Ulferts et al., 2019]. The same is true with regard to the quality of teacher–child relationships in preschool, which have been shown to be predictive of the development of children’s competencies as well (O’Connor and McCartney, 2007; Ahnert and Harwardt, 2008; Sabol and Pianta, 2012; Paes et al., 2023). In view of such findings, research has focused on the characteristics associated with the quality of interactions and relationships. In this regard, previous research has identified predictors of interaction and relationship quality of preschool children, such as structural characteristics, child characteristics, and activity settings (Rudasill et al., 2006; Tietze et al., 2013; Smidt and Embacher, 2023a,b).

The professionalization of preschool teachers is also discussed as an important factor to ensure and improve the quality of interactions and relationships in preschool (Durand et al., 2016; Smidt, 2018; Sanches-Ferreira et al., 2022). This becomes apparent in professionalization models^2^ (e.g., Fröhlich-Gildhoff et al., 2011; Anders, 2012) where preschool teachers’ professional competencies are considered to be predictive of the quality of pedagogical practices in preschools in terms of educational beliefs (e.g., beliefs about how children should be supported), self-regulatory skills (e.g., work engagement), and motivational and emotional aspects (e.g., self-efficacy), along with other characteristics such as vocational knowledge and personality traits. In part, these models were initially developed for the school context (Baumert and Kunter, 2006) and subsequently applied to preschools (Anders, 2012). Previous empirical findings indicate that preschool teachers’ educational beliefs, work engagement, and self-efficacy can influence the quality of interactions and relationships in preschool (e.g., Pianta et al., 2005; Hamre et al., 2008; Penttinen et al., 2020). However, there are also studies revealing quite weak or even a lack of associations (e.g., Hu et al., 2021; Peters et al., 2022).

Most previous research has been conducted outside of Austria and indicates only limited generalizability to specific country contexts such as Austria (e.g., see Love et al., 2003 for a comparison of findings of a US study to other countries). For example, a specific characteristic of the professionalization of preschool teachers in Austria is that they have to complete 5 years of vocational training and do not need an academic degree, as is common in many countries (Smidt, 2018). Against this background, the aim of this study is to investigate associations between preschool teachers’ educational beliefs, work engagement, and self-efficacy with interaction quality and relationship quality in preschools with a focus on Austria. This aim is linked to the intention to obtain recent insights into the predictive importance of preschool teachers’ competencies for the quality of interactions and relationships in Austrian preschools, which may lead to practical implications for improving preschool teachers’ education and training.

## The quality of interactions in preschool

The quality of interactions in preschool can be theoretically framed with ecosystemic approaches (Bronfenbrenner and Morris, 2006) that highlight the preschool class as a microsystem where children and preschool teachers are involved in interactions and activities. Other foundations for interaction quality in preschool include social constructivist approaches based on Vygotsky (Bodrova and Leong, 2018), which emphasize the function of preschool teachers as co-constructors, and domain-specific theories (Wellman and Gelman, 1998), which allow us to focus on specific domains such as language and mathematics (Anders et al., 2012; Smidt and Rossbach, 2016). When asking what comprises “good” quality of interactions, reference can be made to developmentally appropriate practices (Copple and Bredekamp, 2009), according to which children should experience developmentally appropriate interactions and activities covering different domains (e.g., language, mathematics). Preschool teachers are expected to provide enriching pedagogical activities, facilitate social relationships, and ensure healthy and safe care (Tietze et al., 1998; Cryer, 1999; Smidt and Rossbach, 2016). There is no standard method for measuring the quality of interactions, and research findings may differ based on various methodological aspects, including instrumental measurement of quality with specific focal points or level of aggregation with foci on children and/or preschool teachers (e.g., Halle et al., 2010). For instance, some instruments measure interaction quality focusing on specific domains such as literacy or mathematics (e.g., Four Curricular Subscales Extension to the Early Childhood Environment Rating Scale, ECERS-E, Sylva et al., 2011), whereas others measure more global aspects (e.g., Classroom Assessment Scoring System, CLASS Pre-K, Pianta et al., 2008). Furthermore, some instruments focus on the level of the preschool group (e.g., CLASS Pre-K, Pianta et al., 2008), whereas others focus on the level of the specific child (e.g., Individualized Classroom Assessment Scoring System, inCLASS, Downer et al., 2012).

## The quality of relationships in preschool

The quality of relationships in preschool can be framed with the “Conceptual Model of Child-Teacher Relationships” (Pianta et al., 2003), which is based on attachment theory and ecosystemic approaches. A basic assumption is that relationships are marked by the complex interaction of four elements. *Features of individuals* (e.g., gender, self-efficacy) are considered the most basic elements of relationships. *Representational models* can be understood as a “set of feelings and beliefs that has been stored about a relationship that guides feelings, perceptions, and behavior in that relationship” (Pianta et al., 2003, p. 210). *Information exchange processes* can be defined as mutual exchanges and particularly the way in which information is shared between a preschool teacher and a child. With *external influences*, cultural and structural characteristics of the preschool also need to be considered (Pianta et al., 2003).

The “Student-Teacher Relationship Scale” (STRS; Pianta, 2001) is frequently applied as an instrument to capture the quality of relationships in preschools. The STRS covers “closeness” (the degree to which a teacher views a relationship with a child as being friendly and warm), “conflict” (the extent to which a preschool teacher struggles with a child and considers the child to be angered or incalculable), and “dependency” (the degree of seeing the child as demanding help when not necessary and responding severely to separation from the preschool teacher). In many studies, however, only “closeness” and “conflict” are investigated (Verschueren and Koomen, 2021).

## Professional competencies

The professionalization of preschool teachers has been discussed as a key factor to ensure high-quality educational practices in preschool. There are different theoretical concepts and definitions for professionalization (Thole and Polutta, 2011; Smidt et al., 2017). In the current study, we rely on competence-based models of professionalization, which provide a framework to examine the skills and abilities of pedagogues that are relevant to action in practice. According to Anders (2012), professional competencies of preschool teachers comprise professional knowledge (which is not the subject of this study), educational beliefs, self-regulatory skills, and motivational and emotional aspects.

Educational beliefs can be seen as emotional–cognitive traits that influence how one interprets certain situations (filter function), how one acts in those situations (frame function), and whether or how one changes beliefs through new information and experiences (guide function) (Fives and Buehl, 2012). Educational beliefs are multifaceted and can include a preschool teacher’s view on how to support children in their development (e.g., Schmidt and Smidt, 2021), preschool teacher’s educational goals (e.g., Smidt et al., 2015), and preschool teachers’ views on what practices are developmentally appropriate or inappropriate (e.g., Leung, 2012). Educational beliefs are considered to be rather stable but could be modified or rethought based on the preschool teacher’s new content knowledge, experiences, and self-reflective processes, which could be derived from professional training, for instance (Anders, 2012; Fives and Buehl, 2012).

Self-regulatory skills such as work engagement are connected with personal wellbeing and health, which further influence work performance (e.g., Bakker et al., 2014). Work engagement is a positive state of mind toward one’s own work (Schaufeli and Bakker, 2004). Work-related personal compassion and happiness contribute to balanced work engagement among preschool teachers and thus serve as protective factors for wellbeing and health (De Stasio et al., 2020).

Regarding motivational and emotional aspects, self-efficacy is one of the central characteristics of research on early childhood (Tschannen-Moran and Hoy, 2001). Self-efficacy refers to the belief in one’s own capability to perform at a desired level and is related to the confidence of preschool teachers in their own abilities to perform successfully in the classroom. Therefore, self-efficacy impacts preschool teachers’ motivation to (re-)act and affects how much effort they put forth in daily situations and challenges. This also includes feelings of being capable or incapable of offering relevant learning situations for children or supporting children in their development and learning.

## Associations between professional competencies and the quality of interactions and relationships in preschool

Professionalization models point to the importance of preschool teachers’ professional competencies for their pedagogical actions and educational quality (for an overview, see Anders, 2012). In addition, personal characteristics of the preschool teachers, such as teachers’ beliefs, self-efficacy, or work engagement, which are considered professional competencies in this study, can be discussed in the context of the conceptual model of teacher–child relationships described by Pianta et al. (2003). As Pianta et al. (2003) noted, features of individuals are an essential element of teacher–child relationships. For example, teachers’ beliefs and perceptions of children and their role are seen as important in shaping supportive relationships (Myers and Pianta, 2008).

Although associations can be assumed between professional competencies and the quality of interactions and relationships in preschool, research findings thus far have been inconsistent. Regarding teacher beliefs, Pianta et al. (2005) showed that interaction quality was higher among preschool teachers with less teacher-centered and more child-centered beliefs. In interactions with classroom age diversity, preschool teachers’ child-centered beliefs also have a buffering effect on interaction quality (Ansari and Pianta, 2019). Furthermore, Wieduwilt et al. (2023) reported a marginally significant positive association of child-centered beliefs supporting language education embedded in daily routines and an aspect of interaction quality and also a negative association of teacher-directed beliefs supporting additional language programs and another aspect of interaction quality. In contrast to these findings, Hamre et al. (2008) showed that for children with high levels of problem behavior, more authoritarian, teacher-centered beliefs were related to better relationships in terms of less teacher–child conflicts. Other studies found weak or even a lack of associations between preschool teachers’ beliefs and teacher practices, interaction quality and relationship quality (Wilcox-Herzog, 2002; Mashburn et al., 2006; Wen et al., 2011; Peters et al., 2022).

Regarding work engagement, higher work engagement was found to have positive effects on different aspects of interaction quality in kindergarten and elementary schools (Penttinen et al., 2020; Soininen et al., 2023). Furthermore, the job demands–resources model (e.g., Bakker, 2011; Bakker et al., 2014; Bakker and Demerouti, 2017) suggests that work engagement in general has a positive effect on job performance as persons with higher work engagement more often experience positive emotions (e.g., joy, enthusiasm), which help them to expand their thought–action repertoire and to build resources. However, there have been few studies with a focus on work engagement and the quality of interactions (especially at the individual child level) and relationships in preschools.

With regard to self-efficacy, recent studies found positive associations between self-efficacy and aspects of interaction quality in preschool classrooms (Jennings, 2015; Hu et al., 2021; Wolstein et al., 2021). Furthermore, higher levels of preschool teachers’ self-efficacy were associated with higher relationship quality in terms of more closeness and fewer conflicts between teachers and children (Mashburn et al., 2006; Hamre et al., 2008). However, some studies found unexpected or even a lack of associations between self-efficacy and the quality of interactions and relationships (Zee and Koomen, 2016; Hu et al., 2021).

## Preschool education in Austria

While the research reported so far provides valuable insights, its generalizability to preschool education in Austria remains limited. Preschool education in Austria is regulated by the nine federal states in terms of personnel, structures, and technical supervision, while the training of preschool teachers and guidelines on educational work are mainly regulated by the federal government (Hartel et al., 2019). The vocational qualification of preschool teachers regularly takes 5 years and occurs at colleges of higher vocational education and training (BAfEPs). In addition, BAfEPs can offer post-secondary courses for holders of a higher education entrance qualification. The age of prospective teachers at the beginning of the 5-year educational training period is around 14 years, which is low (Hartel et al., 2019). Around 98% of the pedagogical staff working in preschools and similar institutions in Austria is female (Krenn-Wache, 2017). The proportion of male staff is thus about 2%, which is significantly lower than in many other countries (e.g., Denmark, where about 13% of preschool staff are male; Jensen, 2017). Compared to other countries, the proportion of academics among preschool teachers is also low (Smidt et al., 2017; Hartel et al., 2019). Preschools in Austria have their own educational mandate, which is specified in the Nationwide Framework Curriculum for Austrian ECEC Services (Charlotte Bühler Institut, 2009) that defines educational domains (e.g., language, emotions) and conducive pedagogical behavior. Preschools in Austria are usually attended by children from the age of 3 until they start school, with the last year of preschool (the year before starting school) being compulsory for all children since 2009 (Smidt, 2018). Depending on the legal foundations of each federal state, group sizes in the preschools may range from 20 to 25, with one preschool teacher plus at least half an assistant in each group (Hartel et al., 2019). Thus, legally defined staff–child ratios in preschool groups vary among the federal states with ratios, ranging from 1:12 to 1:17 (Schreyer and Oberhuemer, 2017a).

## Study aims

The quality of interactions and relationships that children experience in preschool has been shown to be predictive of children’s competencies and is influenced by different aspects such as structural characteristics and child characteristics. Theoretical frameworks and empirical findings suggest that preschool teachers’ professionalization in terms of educational beliefs, work engagement, and self-efficacy also have predictive importance for the quality of interactions and relationships in preschool. However, there are also some caveats. (1) Research findings are generally inconsistent, with some studies reporting no associations. (2) There is a lack of studies on the situation in preschools in Austria, and international research findings likely have only limited transferability. Therefore, the aim of the present study is to address this research gap by identifying associations between the professional competencies of preschool teachers (educational beliefs, work engagement, and self-efficacy) and the quality of interactions and relationships that children experience in preschool. Child characteristics and structural characteristics served as controls.

## Materials and methods

### Participants

This study used data from the Austrian project “Quality of children’s interactions in preschool,” which was funded by the Austrian Science Fund (FWF). The study focuses primarily on the second wave, for which data collection took place between October and December 2019. The sample comprises 287 children (141 girls) from 89 preschool classes (from 89 randomly selected preschools) in Tyrol, Austria. The children were 3 to 5 years old (*M* = 54.89 months, *SD* = 4.39, minimum = 40.31, maximum = 63.08). Data from 85 preschool teachers (84 female) were collected with paper–pencil questionnaires. The preschool teachers were 38.13 years old on average (*SD* = 11.73) and had *M* = 14.84 years of work experience (*SD* = 10.85).

All preschool teachers had completed vocational training, with 85.88% having completed 4 or 5 years of non-academic training, 11.76% having completed shortened 2-year training (for students with a university entrance qualification), and 2.35% having completed other vocational training. Overall, 35.29% of the preschool teachers had a leading function in the preschool. To comply with ethical guidelines, identifiable information was protected, and data were anonymized to guarantee personal privacy. Furthermore, the participants (parents as representatives for their children) signed an informed consent form.^3^

### Measures

#### Interaction quality

As mentioned in the section “The Quality of Interactions in Preschool,” there are various ways to capture interaction quality, including observation measures focusing on the level of the preschool group and the level of the individual child. An important advantage of measures focusing on the individual child compared to measures focusing on the level of the preschool group is that variations in the experiences of individual children regarding their interaction with their preschool teacher and peers can be assessed (Chien et al., 2010; Smidt and Rossbach, 2016). Therefore, interaction quality was measured at the individual child level with the observation tool “Individualized Classroom Assessment Scoring System” (inCLASS, Downer et al., 2012). This tool measures children’s interactions with teachers (e.g., the child’s communication with teachers), peers (e.g., the child’s experience of positive emotions with peers), and tasks (the degree to which the child is actively involved in tasks and activities) (Downer et al., 2010). Based on previous research (Downer et al., 2010; Slot et al., 2015; von Suchodoletz et al., 2015; Bohlmann et al., 2019; Smidt and Embacher, 2021), the 10 inCLASS dimensions were grouped into the following factors: *teacher interactions* (α = 0.85), which includes positive engagement with the teacher and teacher communication, *peer interactions* (α = 0.85), which includes peer sociability, peer communication, and peer assertiveness, *task orientation* (α = 0.58), which includes task engagement and self-reliance, and *conflict interactions* (α = 0.74), which includes teacher conflict, peer conflict, and behavior control (reverse coded). See Table 1 for descriptive statistics and Table 2 for intercorrelations. Except for task orientation, the internal consistencies of the factors are acceptable to good (Nunnally, 1978).

**TABLE 1:** Descriptive results.

	*N*	%	*M*	*SD*
**Interaction quality**				
Teacher interactions	287		2.16	0.91
Peer interactions	287		2.98	0.88
Task orientation	287		4.43	0.81
Conflict interactions	287		1.26	0.35
**Relationship quality**				
Teacher–child closeness	275		33.25	6.08
Teacher–child conflict	273		11.55	6.07
**Professional competencies**				
Beliefs on self-education	85		4.01	0.62
Beliefs on co-construction	85		4.55	0.43
Beliefs on instruction	85		2.62	0.77
Work engagement	85		4.76	0.87
Self-efficacy	85		4.33	0.51
**Child characteristics**				
Gender of the children	287			
girls	141	49.13		
boys	146	50.87		
Age of the children (in months)	287		54.89	4.39
Language skills (*T*-value) of the children	262		47.88	9.49
Child personality types	282			
Overcontrollers	69	24.47		
Undercontrollers	88	31.21		
Resilients	125	44.33		
**Structural characteristics**				
Child–staff ratio	80		7.47	2.06
Work experience of the preschool teachers	85		14.84	10.85
Number of children with immigration background per preschool class	84		3.76	5.00
Adequate equipment in the preschool class	85		2.24	0.42

*N* = sample size, % = percent, *M* = mean, *SD* = standard deviation.

**TABLE 2:** Intercorrelations between study variables.

	1	2	3	4	5	6	7	8	9	10	11	12	13	14	15	16	17	18	19	20
1	–																			
2	0.24***	–																		
3	0.24***	0.45***	–																	
4	−0.14*	0.04	−0.12*	–																
5	0.23***	0.07	0.09	−0.07	–															
6	−0.10^#^	−0.08	−0.12^#^	0.28***	−0.57***	–														
7	−0.06	0.08	−0.05	0.07	0.03	−0.04	–													
8	0.02	0.11^#^	−0.06	0.11^#^	0.09	−0.18**	0.25***	–												
9	−0.08	0.04	0.16**	−0.08	−0.05	0.01	−0.30***	0.12^#^	–											
10	0.08	0.11^#^	0.08	0.16**	0.34***	−0.20***	0.22***	0.40***	−0.02	–										
11	−0.13*	−0.01	0.04	0.03	0.15*	−0.19**	0.04	0.03	−0.01	0.33***	–									
12	−0.07	−0.02	0.10^#^	0.21***	−0.11^#^	0.06	−0.13*	−0.09	−0.03	0.00	0.04	–								
13	0.05	0.29***	0.16**	0.01	0.08	−0.15*	0.00	0.08	0.12*	0.05	−0.06	0.01	–							
14	0.14*	0.18**	0.17**	−0.14*	0.15*	−0.19**	−0.10	0.03	0.02	−0.03	−0.04	−0.03	−0.03	–						
15	−0.18**	−0.19**	−0.14*	−0.02	−0.39***	0.04	−0.06	−0.01	0.00	−0.07	0.02	0.09	−0.01	−0.30***	–					
16	0.00	0.00	−0.05	0.17**	0.03	0.27***	0.03	0.01	−0.12^#^	0.03	0.06	0.06	−0.12*	−0.04	− 0.38***	–				
17	0.15^#^	0.16**	0.16**	−0.14*	0.31***	−0.28***	0.03	0.00	0.11^#^	0.03	−0.08	−0.13*	0.12*	0.29***	−0.51***	−0.60***	–			
18	−0.02	0.05	−0.03	−0.06	−0.19**	0.06	−0.05	−0.04	0.16*	−0.31***	−0.04	−0.03	0.11^#^	0.08	−0.04	−0.08	0.11^#^	–		
19	−0.02	−0.01	0.04	−0.03	−0.16**	−0.04	0.03	−0.07	0.03	−0.17**	0.22***	0.04	0.05	−0.14*	0.07	−0.03	−0.03	0.14*	−	
20	−0.15*	−0.13*	−0.10*^#^*	0.00	−0.14*	0.09	0.08	−0.13*	0.05	−0.09	0.02	0.02	−0.03	−0.31***	0.16**	−0.07	−0.07	0.18**	0.31***	−
21	0.10	0.00	−0.02	0.06	0.05	−0.05	−0.01	0.18**	−0.15*	0.09	−0.05	−0.07	0.09	−0.12*^#^*	0.00	0.03	−0.03	−0.06	0.20**	0.04

Pearson’s correlations were computed. 1 = teacher interactions, 2 = peer interactions, 3 = task orientation, 4 = conflict interactions, 5 = teacher–child closeness, 6 = teacher–child conflict, 7 = beliefs on self-education, 8 = beliefs on co-construction, 9 = beliefs on instruction, 10 = work engagement, 11 = self-efficacy, 12 = gender of the children (0 = girls, 1 = boys), 13 = age of the children (in months), 14 = language skills of the children, 15 = overcontrollers, 16 = undercontrollers, 17 = resilients, 18 = child–staff ratio, 19 = work experience of the preschool teachers, 20 = number of children with immigration background per preschool class, 21 = adequate equipment in the preschool class.

^#^*p* < 0.10, *p < 0.05, **p < 0.01, ***p < 0.001.

On one observation day (typically from 8 a.m. to 12 p.m.), up to four children from one preschool class were observed with up to four alternating observation cycles in different situations (e.g., free play, mealtime, planned activity). According to the manual, four cycles per child (a total of 16 observations for four children) can be completed in a 4-h visit (Downer et al., 2012). One cycle lasts 15 min, with 10 min for observing children and note-taking followed by 5 min for scoring in consultation with the manual (Downer et al., 2012). To determine scores, each dimension receives a code of 1 to 7 indicating a low (1 or 2), medium (3 to 5), or high (6 or 7) level, and data from each observation cycle are averaged to obtain a final score (Downer et al., 2010).

Data collection was carried out by 13 student assistants (students of educational science and psychology), who completed 2 days of training by a certified inCLASS trainer, which included a reliability test. In the first wave of the study, double coding was conducted to examine inter-rater reliability. Intraclass correlation coefficients (ICCs) of the single domains ranged between 0.75 and 0.95, indicating excellent inter-rater agreement (Cicchetti, 1994; see Smidt and Embacher, 2023a for information on the first study wave).

#### Relationship quality

Preschool teachers rated their perception of their relationship with a particular child using a short form of the “Student-Teacher Relationship Scale” (STRS) (Pianta, 2001), which includes 15 items rated on a five-point scale ranging from 1 (“definitely does not apply”) to 5 (“definitely applies”) (Pianta, 1992). The German version used here was also part of the NUBBEK study (Tietze et al., 2015). The *closeness* subscale consists of eight items (α = 0.87, e.g., “I share an affectionate, warm relationship with this child”), and the *conflict* subscale consists of seven items (α = 0.89, e.g., “This child easily becomes angry at me”). For each subscale, a sum score of the individual items was calculated (Pianta, 2001).

#### Professional competencies

Preschool teachers’ educational beliefs regarding their support of children were measured with a scale that was used in a German study (Schmidt and Smidt, 2021). The scale consists of 12 items assigned to subscales: *beliefs on self-education* (four items, α = 0.69., e.g., “When supporting children, it is important that the preschool teacher interfere as little as possible”), *beliefs on co-construction* (four items, α = 0.52, e.g., “When supporting children, it is important that the children are encouraged by the preschool teacher to find their own solutions”), and *beliefs on instruction* (four items, α = 0.79, e.g., “When supporting children, it is important that the children are taught a lot by the preschool teacher”). Each item was rated on a five-point scale ranging from 1 (“strongly disagree”) to 5 (“strongly agree”) (Schmidt and Smidt, 2021).

In contrast to the other study variables, preschool teachers’ educational beliefs regarding their support of children were partially measured during the first study wave (from April to June 2019), which took place about half a year before the second wave. Due to time and economic reasons, preschool teachers participating in the first wave of the study (71.76% of the preschool teachers in the current sample) did not have to rate their beliefs on self-education, co-construction, and instruction again in the second wave. In these cases, data from the first wave were used. For newly participating preschool teachers (28.24% of the sample), data on educational beliefs were collected during the second wave (from October to December 2019).

Preschool teachers’ *work engagement* was assessed with the “Utrecht Work Engagement Scale” (UWES-9) (Schaufeli et al., 2006). This short form consists of nine items (α = 0.94, e.g., “At my work, I feel bursting with energy”), which are rated on a seven-point scale ranging from 0 (“never”) to 6 (“always”) (Schaufeli et al., 2006). Preschool teachers’ *self-efficacy* was captured with the “Kurzskala zur Erfassung allgemeiner Selbstwirksamkeitserwartungen (short scale for measuring general self-efficacy beliefs)” (ASKU) (Beierlein et al., 2013), which measures general self-efficacy beliefs with three items (α = 0.86, e.g., “I can rely on my own abilities in difficult situations”) that are rated on a five-point scale ranging from 1 (“strongly disagree”) to 5 (“strongly agree”).

#### Child characteristics and structural characteristics

Based on previous study findings (e.g., Justice et al., 2008; Rudasill and Rimm-Kaufman, 2009; Downer et al., 2012; Linberg and Kluczniok, 2020; Ramirez and Linberg, 2022; Smidt and Embacher, 2023a,b), child characteristics (age, gender, language skills, personality types) and structural characteristics (child–staff ratio, preschool teachers’ work experience, number of children with immigration background per preschool class, and adequate equipment in the preschool class) were considered as control variables. The *age* of the children and their *gender* were captured through paper–pencil interviews or telephone interviews with their parents. Children’s *language skills* were measured with a mean score (*T*-value) of three subtests (understanding sentences, morphological rule formation, and phonological working memory, α = 0.73) of the “Sprachentwicklungstest für drei- bis fünfjährige Kinder (language development test for 3 to 5-year-old children)” (SETK 3–5) (Grimm, 2015).

*Child personality* was measured using the “Fünf Faktoren Fragebogen für Kinder−Kurzform (Five Factor questionnaire for children – short version)” (FFFK-K) (Asendorpf, 2007), which was developed within the framework of the Socio-Economic Panel (SOEP). Preschool teachers assessed children’s personality with 10 bipolar items (originally rated on an 11-point scale ranging from 0 to 10), which were assigned to the following factors: neuroticism (α = 0.71; e.g., worried−calm), extraversion (α = 0.85; e.g., talkative−quiet), intellect (α = 0.73; e.g., interested−uninterested), agreeableness (α = 0.73; e.g., gentle−stubborn), and conscientiousness (α = 0.60; e.g., orderly−disorderly) (Asendorpf, 2007; see Smidt and Embacher, 2023a for further information on the use of the questionnaire in the first study wave).

Three personality types have been shown to be predictive of several social outcomes and problem behaviors among children: *resilients*, *overcontrollers*, and *undercontrollers* (e.g., Asendorpf and van Aken, 1999; Hart et al., 2003; van den Akker et al., 2013). To derive these types, we followed the method of Asendorpf et al. (2001) and conducted a two-step clustering procedure (Ward, followed by k-means) (for a detailed description, see Smidt and Embacher, 2023a). The “Big Five” cluster profiles show that resilients are characterized by below-average scores on neuroticism and above-average scores on extraversion, intellect, agreeableness, and conscientiousness. Overcontrollers are described by above-average scores on neuroticism and below-average scores on extraversion, intellect, agreeableness, and conscientiousness. Undercontrollers score above average on extraversion and below average on neuroticism, intellect, agreeableness, and conscientiousness.

Referring to previous studies (e.g., Asendorpf et al., 2001; Barbaranelli, 2002), a double-cross validation was applied to evaluate the consistency of the three-cluster solution across 10 different subsamples (see Smidt and Embacher, 2023a). The median κ-value was κ = 0.81, which is considered acceptable (Asendorpf et al., 2001). Regarding structural characteristics, the *child–staff ratio*, preschool teachers’ *work experience*, and the *number of children with an immigration background per preschool class* were determined from the information provided by the preschool teachers. The child–staff ratio was calculated by dividing the number of children in the preschool group by the number of staff (preschool teachers and assistants) in the group. The resulting number indicates how many children are cared for per staff member. Work experience was measured by asking preschool teachers how many years in total they had been working in the profession. The number of children with an immigration background per preschool class was computed on the basis of the information provided by the preschool teachers. Preschool teachers’ perception of *adequate equipment in the preschool class* (e.g., sufficient writing materials, books) was captured with seven items (α = 0.65) from a scale used in the German BiKS study (based on the Home Observation for Measurement of the Environment, Caldwell and Bradley, 1984). Preschool teachers had three answer options (The following tools are available so often that. 1 = some children can play with it, 2 = about half of all children can play with it, 3 = almost all children can play with it).

### Data analyses

To study associations between professional competencies and aspects of both interactional quality and relationship quality, multiple hierarchical regression analyses were conducted with the four factors of the inCLASS (*teacher interactions, peer interactions, task orientation*, and *conflict interactions*) and the two subscales of the STRS (*teacher–child closeness* and *teacher–child conflict*) as dependent variables. Preschool teachers’ beliefs on self-education, co-construction, and instruction, as well as work engagement and self-efficacy, served as predictors (step 1). In the next step, child characteristics (gender, age, language skills, and personality types) and structural characteristics were considered as control variables (step 2). Robust standard errors were calculated in Stata as the children were nested in preschool classes (Williams, 2000). Due to missing values, the sample sizes in the regression analyses were reduced to *N* = 231 (see Table 3) and *N* = 225 (see Table 4).

**TABLE 3:** Prediction of interaction quality through professional competencies.

	Teacher interactions			Peer interactions			Task orientation			Conflict interactions		
**Predictors**	***B***	***SE***	**β**	***B***	***SE***	**β**	***B***	***SE***	**β**	***B***	***SE***	**β**
*Step 1*												
**Professional competencies**												
Beliefs on self-education	−0.19	0.15	−0.12	0.06	0.11	0.04	−0.01	0.09	−0.01	−0.02	0.04	−0.04
Beliefs on co-construction	0.06	0.21	0.03	0.04	0.17	0.02	−0.46	0.19	−0.24*	0.01	0.05	0.02
Beliefs on instruction	−0.14	0.10	−0.12	0.08	0.09	0.07	0.24	0.07	0.23**	−0.01	0.02	−0.02
Work engagement	0.18	0.12	0.16	0.10	0.09	0.10	0.04	0.08	0.04	0.07	0.02	0.19**
Self-efficacy	−0.28	0.20	−0.16	−0.06	0.12	−0.03	0.18	0.14	0.12	−0.02	0.04	−0.03
*R*^2^ (*R*^2^ adjusted)	0.05	(0.03)		0.02	(−0.00)		0.09	(0.07)		0.03	(0.01)	
*F*	0.85			0.88			3.51**			2.11^#^		
*Step 2*												
**Professional competencies**												
Beliefs on self-education	−0.21	0.14	−0.14	0.08	0.10	0.06	0.04	0.09	0.03	−0.01	0.04	−0.02
Beliefs on co-construction	0.03	0.20	0.01	0.06	0.18	0.03	−0.48	0.17	−0.25**	0.00	0.05	0.01
Beliefs on instruction	−0.14	0.09	−0.11	0.03	0.12	0.03	0.28	0.08	0.26**	0.01	0.02	0.02
Work engagement	0.17	0.13	0.15	0.07	0.10	0.07	−0.03	0.08	−0.03	0.06	0.03	0.17*
Self-efficacy	−0.24	0.20	−0.14	−0.01	0.16	−0.01	0.24	0.16	0.16	0.00	0.04	0.00
**Child characteristics**												
Gender (0 = girls, 1 = boys)	−0.19	0.10	−0.10^#^	0.01	0.10	0.01	0.17	0.09	0.11^#^	0.12	0.04	0.19**
Age (in months)	0.00	0.01	0.01	0.06	0.01	0.30***	0.02	0.01	0.09	−0.00	0.01	−0.06
Language skills	0.00	0.01	0.04	0.01	0.01	0.15^#^	0.01	0.01	0.14*	−0.01	0.00	−0.15^#^
Overcontrollers*^a^*	−0.43	0.14	−0.20**	−0.23	0.17	−0.11	−0.25	0.14	−0.14^#^	−0.04	0.05	−0.05
Undercontrollers*^a^*	−0.11	0.14	−0.06	−0.03	0.14	−0.02	−0.07	0.11	−0.04	0.10	0.05	0.15*
**Structural characteristics**												
Child–staff ratio	0.01	0.06	0.03	0.00	0.04	0.01	−0.04	0.04	−0.11	0.01	0.01	0.08
Work experience	0.01	0.01	0.10	0.00	0.01	0.04	0.01	0.01	0.08	−0.00	0.00	−0.13^#^
Number of children with immigration background per preschool class	−0.04	0.03	−0.14	−0.01	0.02	−0.01	−0.03	0.02	−0.14	0.00	0.01	0.05
Adequate equipment in the preschool class	0.00	0.19	0.00	−0.06	0.26	−0.03	0.05	0.16	0.03	0.09	0.06	0.11
*R*^2^ (*R*^2^ adjusted)	0.13	(0.07)		0.16	(0.10)		0.19	(0.13)		0.15	(0.09)	
*F*	1.80^#^			3.88***			3.72***			2.27*		
Δ*R*^2^	0.08*			0.14***			0.10***			0.12**		

*N* = 231; *B* = unstandardized regression coefficient; *SE* = clustered robust standard error; β = standardized regression coefficient.

^*a*^ The reference category is resilients.

^#^*p* < 0.10, *p < 0.05, **p < 0.01, ***p < 0.001.

**TABLE 4:** Prediction of relationship quality through professional competencies.

	Teacher–child closeness			Teacher–child conflict		
**Predictors**	***B***	***SE***	**β**	***B***	***SE***	**β**
*Step 1*						
**Professional competencies**						
Beliefs on self−education	−0.03	1.05	−0.00	0.25	1.05	0.03
Beliefs on co−construction	0.30	0.96	0.02	−1.70	0.86	−0.12^#^
Beliefs on instruction	−0.81	0.52	−0.10	0.66	0.55	0.08
Work engagement	2.48	0.71	0.35***	−0.42	0.65	−0.06
Self−efficacy	0.36	0.97	0.03	−2.27	1.02	−0.21*
*R*^2^ (*R*^2^ adjusted)	0.15	(0.13)		0.09	(0.07)	
*F*	3.68**			1.77		
*Step 2*						
**Professional competencies**						
Beliefs on self-education	−0.30	0.88	−0.03	0.04	0.80	0.00
Beliefs on co-construction	0.48	1.06	0.03	−2.11	0.94	−0.15*
Beliefs on instruction	−0.72	0.45	−0.09	1.00	0.41	0.13*
Work engagement	1.58	0.70	0.23*	−0.27	0.59	−0.04
Self-efficacy	1.70	0.94	0.15^#^	−2.30	1.01	−0.21*
**Child characteristics**						
Gender (0 = girls, 1 = boys)	−0.91	0.57	−0.08	0.20	0.52	0.02
Age (in months)	0.18	0.08	0.14*	−0.14	0.09	−0.11
Language skills	0.03	0.04	0.05	−0.10	0.04	−0.17*
Overcontrollers*^a^*	−6.04	1.06	−0.44***	1.39	0.95	0.10
Undercontrollers*^a^*	−1.63	0.77	−0.13*	4.45	0.88	0.36***
**Structural characteristics**						
Child–staff ratio	−0.42	0.24	−0.15^#^	0.21	0.25	0.07
Work experience	−0.04	0.04	−0.07	–0.04	0.04	−0.08
Number of children with immigration background per preschool class	0.06	0.14	0.04	0.13	0.10	0.09
Adequate equipment in the preschool class	0.52	1.00	0.04	0.74	0.70	0.05
*R*^2^ (*R*^2^ adjusted)	0.36	(0.32)		0.29	(0.24)	
*F*	7.04***			5.58***		
Δ*R*^2^	0.21***			0.20***		

*N* = 225; *B* = unstandardized regression coefficient; *SE* = clustered robust standard error; β = standardized regression coefficient.

^*a*^The reference category is resilients.

^#^*p* < 0.10, *p < 0.05, **p < 0.01, ***p < 0.001.

## Results

The descriptive results are presented in Table 1, and intercorrelations of the study variables are shown in Table 2. Tables 3, 4 present detailed results of the regression analyses (step 1 and step 2), including unstandardized and standardized regression coefficients, clustered robust standard errors, *F*-test, coefficient of determination (*R*^2^), adjusted *R*^2^, and change in *R*^2^.

### Interaction quality

The results of *teacher interactions* showed no significant effects of teachers’ beliefs on self-education, co-construction and instruction, work engagement, and self-efficacy. The gender of the children (only *p* < 0.10) and classification as an overcontroller were negatively related to *teacher interactions*. Regarding *peer interactions*, no significant effects of teachers’ beliefs on self-education, co-construction and instruction, work engagement, and self-efficacy were found. However, the language skills of the children (only *p* < 0.10) and their age were positively related to *peer interactions*. The findings of *task orientation* showed that preschool teachers’ beliefs on co-construction were negatively related to *task orientation*, whereas teachers’ beliefs on instruction were positively related to *task orientation*. However, there were no effects of preschool teachers’ beliefs on self-education, work engagement, and self-efficacy on *task orientation*. Language skills of the children were positively related to task orientation, and gender (only *p* < 0.10) tended to have a positive effect on *task orientation*. Furthermore, classification as an overcontroller (only *p* < 0.10) tended to be negatively related to *task orientation*. Regarding *conflict interactions*, positive effects of work engagement were found. Preschool teachers’ beliefs on self-education, co-construction and instruction, and self-efficacy were not related to *conflict interactions*. Gender and classification as an undercontroller had positive effects on *conflict interactions*. Furthermore, language skills (only *p* < 0.10) and work experience (only *p* < 0.10) tended to be negatively related to *conflict interactions*.

### Relationship quality

The findings of *teacher–child closeness* showed a positive effect of preschool teachers’ work engagement. Furthermore, self-efficacy (only *p* < 0.10) tended to be positively related to *teacher–child closeness*. Preschool teachers’ beliefs on self-education, co-construction, and instruction were not related to *teacher–child closeness.* The age of the children was positively related to *teacher–child closeness.* Moreover, classification as an overcontroller or undercontroller was negatively related to *teacher–child closeness*, and the child–staff ratio (only *p* < 0.10) tended to be negatively related to *teacher–child closeness.* The results of *teacher–child conflict* showed a negative effect of teachers’ beliefs on co-construction and a positive effect of teachers’ beliefs on instruction. In addition, teachers’ self-efficacy was negatively related to *teacher–child conflict.* There were no effects of teachers’ beliefs on self-education and work engagement on *teacher–child conflict.* Language skills of the children were negatively related to *teacher–child conflict*, and a classification as an undercontroller was positively related to *teacher–child conflict.*

## Discussion

Relying on a framework of professionalization, the present study investigated associations between professional competencies of preschool teachers in terms of educational beliefs, work engagement, self-efficacy, and the quality of interactions and teacher–child relationships in preschools in Austria. After controlling for child characteristics and structural characteristics, mixed findings were obtained. There were some statistically significant or tendentially significant associations with task orientation, conflict interactions, teacher–child closeness, and teacher–child conflict but no associations with peer interactions.

### Interaction quality

#### Teacher interactions

We did not find statistically significant associations between preschool teachers’ beliefs on self-education, co-construction and instruction, and teacher interactions. Although educational beliefs are considered to filter, frame, and guide pedagogical activities (Fives and Buehl, 2012), it must be also noted that research on educational beliefs and interaction quality is not consistent with studies revealing no influence of preschool teachers’ educational beliefs on interactional processes in preschools (Kluczniok and Roßbach, 2014). In this regard, the results of the present study fit with the inconsistent research landscape. We also did not find associations between preschool teachers’ work engagement, self-efficacy, and teacher interactions. This pattern of findings deviates from previous research, which found positive associations between preschool teachers’ work engagement (Penttinen et al., 2020; Soininen et al., 2023) and self-efficacy (Jennings, 2015; Hu et al., 2021; Wolstein et al., 2021) and the quality of interactions in preschools. An explanation for the null findings may lie in the inCLASS, which primarily focuses on children’s interactions with preschool teachers and not vice versa as many other instruments focusing on interaction quality do (Schmidt et al., 2018). This could probably explain why no associations were found.

#### Peer interactions

We did not find any associations between preschool teachers’ educational beliefs, work engagement, self-efficacy, and the quality of peer interactions. It could be argued that null findings are probably not surprising because children’s interactions with peers are in focus rather than interactions with preschool teachers. Furthermore, reference could be made to the specificities of the inCLASS, which focuses on the children’s perspective on interaction quality and less on preschool teachers. However, this does not mean that preschool teachers do not play an important role in fostering children’s interaction with peers. Research suggests that preschool teachers should use scaffolding (based on Vygotsky; see Bodrova and Leong, 2018) to facilitate children’s interactions with peers (Acar et al., 2017) and should engage children in cognitively stretching “high-yield” activities (Bruner, 1980; Kontos and Wilcox-Herzog, 1997) to promote peer interactions (Smidt and Embacher, 2020). Previous findings from Austria indicate that the potential in this respect has not yet been fully tapped (Smidt and Embacher, 2020).

#### Task orientation

Preschool teachers’ endorsement of instruction was lower on average compared with self-education and co-construction, as Table 1 illustrates (see Schmidt and Smidt, 2021 and Mackowiak et al., 2022 for similar findings). However, it was positively related to children’s task orientation, whereas educational beliefs on co-construction revealed a negative association with task orientation. No effects were found for beliefs on children’s self-education. The positive effect of beliefs on instruction is reasonable because it can be assumed that an endorsement of instruction corresponds with preschool teacher-directed activities with clear learning goals (Schmidt and Smidt, 2021). Preschool teacher-led activities were found to be positively related to the development of children’s academic skills (de Haan et al., 2014; Goble and Pianta, 2017), and previous research also indicates that preschool teacher-led activities may reduce children’s off-task behavior (Rimm-Kaufman et al., 2005), a finding that fits with the present study. This is also true for the null finding regarding the beliefs on self-education, which corresponds with the expectation that preschool teachers endorsing self-education would tend to be reserved in practicing teacher-directed activities with clear learning goals (Schmidt and Smidt, 2021).

Some researchers suggest that a strong focus on children’s self-education and reservation of preschool teachers can be an issue because concerns have been expressed that the children who are most in need of support, targeted stimulation, and active assistance could be disadvantaged (Grell, 2010). The interpretation of the negative association between co-construction beliefs and children’s task orientation is much less clear as one would expect the opposite pattern of results (Winsler and Carlton, 2003; Bodrova and Leong, 2018). An explanation may lie in a lack of clarity on the part of the preschool teachers on how co-construction can be implemented to support children’s task orientation. Some research has revealed a high overlap (*r* = 0.59) between beliefs on co-construction and self-education (Schmidt and Smidt, 2021), whereas other findings suggest that preschool teachers do not rely on co-construction but on self-education (Mackowiak et al., 2022). These somewhat “hazy” findings might indicate a conceptual ambiguity of co-construction and self-education that preschool teachers probably face. Findings showing that cognitively stimulating learning support for children by preschool teachers is hardly implemented in everyday preschool life (Schmidt and Smidt, 2021) can also be interpreted in this direction.

We did not find any associations between preschool teachers’ work engagement and self-efficacy and the quality of interactions in terms of children’s task orientation. These patterns of findings deviate from previous research, which found positive associations between preschool teachers’ work engagement and interaction quality (Penttinen et al., 2020; Soininen et al., 2023) and preschool teachers’ self-efficacy and interaction quality (Jennings, 2015; Hu et al., 2021; Wolstein et al., 2021). However, in all of these studies, interaction quality was examined with the Classroom Assessment Scoring System (CLASS; Pianta et al., 2008), which also comprises interaction quality in terms of time on tasks (classroom organization) but focuses much more strongly on the preschool teacher. Thus, reasons for the lack of findings may lie in the measurement of the inCLASS.

#### Conflict interactions

Regarding conflict interactions, the only statistically significant association was found with preschool teachers in terms of work engagement. There were no statistically significant findings regarding preschool teachers’ beliefs and self-efficacy. The most obvious explanation for the extensive lack of associations is probably the small variance of the conflict interaction domain (see Table 1). There is simply very little variance that can be explained at all. Indeed, the inCLASS factor conflict interactions is viewed critically due to the lack of variance and other measurement problems (von Suchodoletz et al., 2015; Slot and Bleses, 2018; Smidt and Embacher, 2021). There is one exception: we found a positive association between work engagement and conflict interactions (higher extent of conflicts). One possible explanation could involve “absorption,” which is a characteristic of work engagement and means that people are immersed in their work (Bakker, 2011). The findings from our study could imply that preschool teachers with higher work engagement are probably less aware of the (few) conflictive interactions of the children with their peers, with themselves, and in terms of difficulties in behavior control as they are immersed and engaged in their work and different tasks. This should be explored in more detail in future studies.

### Relationship quality

#### Teacher–child closeness

Although educational beliefs of teachers have been considered to be important for the formation of teacher–student relationships (Myers and Pianta, 2008), empirical research is sparse and provides no clear evidence for associations between preschool teachers’ educational beliefs and relationship quality (Mashburn et al., 2006; Hamre et al., 2008). The present study points in the same direction since preschool teachers’ professional competencies in terms of beliefs on self-education, co-construction, and instruction did not predict teacher–child closeness. One explanation for the lack of findings in the present study and in previous research may lie in the nature of educational beliefs, which have been referred to as a “messy” construct (Fives and Buehl, 2012, p. 471) because they are difficult to define and conceptualize.

Preschool teachers’ work engagement was positively associated with teacher–child closeness. Although referring to interactions instead of relations, previous research indicating positive associations between work engagement and interaction quality between preschool teachers and children (Penttinen et al., 2020; Soininen et al., 2023) could probably be tentatively connected to the findings of the present study. Following the job demands–resources model (e.g., Bakker, 2011; Bakker et al., 2014; Bakker and Demerouti, 2017), associations can also be theoretically expected as it is assumed that higher work engagement corresponds with positive emotions such as joy and enthusiasm, which may positively affect relationships with children. However, empirical findings on relations between preschool teachers’ work engagement and teacher–child closeness seem to be sparse. In addition, preschool teachers’ self-efficacy had a significant positive association with teacher–child closeness (only *p* < 0.10). This finding is generally in line with previous research (Mashburn et al., 2006; Hamre et al., 2008) and underpins the still quite limited empirical findings on the importance of preschool teachers’ self-efficacy for the quality of teacher–child relationships (e.g., Chen and Phillips, 2018).

#### Teacher–child conflict

A higher endorsement of educational beliefs on instruction corresponded with a perception of preschool teacher–child relationships as more conflicted. A higher endorsement of beliefs on co-construction corresponded with a perception of these relationships as less conflicted. One explanation could be that resentment and resistance among children could arise from an endorsement of instruction, which corresponds with teacher-directed activities in which the child has little opportunity to have a voice (Schmidt and Smidt, 2021). This resentment and resistance might be manifested in preschool teachers’ perceptions of relationships with children as more conflicted. If this interpretation is true, the effect of beliefs on co-construction also seems reasonable because in educational practice, children might be more actively involved, they might have more opportunities to have a voice, and preschool teachers might be in a dialogue with the children and provide them with targeted assistance (“scaffolding”) (Bodrova and Leong, 2018; Schmidt and Smidt, 2021).

There were no significant associations between preschool teachers’ work engagement and teacher–child conflict. This null finding seems surprising because according to the job demands–resources model (e.g., Bakker, 2011; Bakker et al., 2014; Bakker and Demerouti, 2017), one would expect that high work engagement, which is related to positive emotions such as joy and enthusiasm, would lead to less conflictive relationships between preschool teachers and children, at least in the perception of the preschool teacher. In any case, this finding cannot be readily explained and would need further investigation in additional studies. A different picture emerges with regard to preschool teachers’ self-efficacy, which was negatively related to teacher–child conflict (i.e., higher self-efficacy led to less conflicted relationships between preschool teachers and children in the perception of preschool teachers). This finding fits with previous research where the same pattern of results was reported (Hamre et al., 2008). It also adds additional evidence to the research on preschool teachers’ self-efficacy for the quality of teacher–child relationships (e.g., Chen and Phillips, 2018).

### Control variables

It is important to note that the findings on associations between different components of professional competencies, interaction quality, and relationship quality hold after accounting for powerful control variables, particularly in terms of child characteristics that are related to aspects of interaction quality and/or relationship quality. These findings generally correspond with previous research (e.g., Rudasill and Rimm-Kaufman, 2009; Booren et al., 2012; Slot and Bleses, 2018; Smidt and Embacher, 2023a). However, structural characteristics (see Mashburn et al., 2006; Slot et al., 2015; Smidt and Embacher, 2023b for further findings) were shown to be relatively poor predictors of interaction quality and relationship quality.

### Study limitations and implications for research and practice

When interpreting the results, several limitations must be taken into account. First, it must be considered that interaction quality was assessed at the individual child level with the inCLASS. Although the inCLASS has the advantage of focusing on the interactional experiences of individual children, a drawback is the narrow focus on the preschool teachers and their pedagogical activities. If interaction quality had been captured at the preschool class level with a stronger focus on preschool teachers, some deviating findings might have emerged. To include preschool teachers’ perspectives in the study, the STRS was used to assess teacher–child relationships. Second, preschool teachers’ self-efficacy was captured as general self-efficacy, which is somewhat different from other studies that focused more on teaching self-efficacy (e.g., Mashburn et al., 2006; Hu et al., 2021). However, it is assumed that general self-efficacy beliefs have a tendency to “spill over” into specific situations (e.g., Chen et al., 2001). Third, ceiling effects cannot be excluded as the expression of several professional competencies (e.g., beliefs on co-construction and self-efficacy) was relatively high. High levels in respect to beliefs on co-construction can be explained by the high value placed on co-construction in the Nationwide Framework Curriculum for Austrian ECEC Services (Charlotte Bühler Institut, 2009). High levels of preschool teachers’ self-efficacy have also been found in other studies (e.g., Smidt et al., 2018). Fourth, parts of the teachers’ beliefs were measured about half a year earlier due to the study design. The beliefs collected half a year later were used due to the better response (thus achieving a larger number of cases). As the time frame consisted of only a few months, and educational beliefs can be considered relatively stable (Fives and Buehl, 2012), it is assumed that this should not impact the results. Fifth, some measures had low reliability (e.g., task orientation α = 0.58; beliefs on co-construction α = 0.52), which is possible due the low number of items among these subscales (Cortina, 1993). Although low reliability may not be an obstacle for the usage of scales in every case, it has to be considered when interpreting findings as low reliability can attenuate associations between variables (Schmitt, 1996). Sixth, a limitation is that the considered control variables may be conceptualized differently depending on the study. For example, work experience may refer to the time spent in the current preschool or to the duration of experience across different workplaces. Similarly, the child–staff ratio may refer to preschool teachers and assistant staff or to preschool teachers only. This makes it difficult to compare the results with those of other studies. Seventh, it has to be taken into account that data were collected within one federal state of Austria. As the preschool system in Austria differs from other countries (see the section on “Preschool education in Austria”), transferability to other countries’ contexts will probably be limited. When comparing the current study findings with previous ones, it also has to be taken into account that not only different cultural contexts (Pastori and Pagani, 2017) but also different methodological procedures (e.g., different measurement instruments) with associated methodological problems (e.g., problems with factorial validity, see Smidt and Embacher, 2023c) may influence study results and make comparability more difficult. Despite these limitations, the study provides relevant findings that can be integrated into the international research context.

Although the current findings are somewhat mixed and not always easy to interpret, the study shows that aspects of professional competencies are predictive of the quality of interactions and relationships in preschool, even after consideration of relevant control variables. This leads to several implications, especially in the context of the vocational training and professional development of preschool teachers. In light of the study results, it seems to be relevant that preschool teachers become aware of the importance of their professional competencies for their pedagogical activities and have the possibility to reflect them (e.g., regarding their beliefs on the support of children). Particular focus should be placed on beliefs on co-construction and their implementation in practice. Despite preschool teachers highly agreeing with beliefs on co-construction, the study results suggest that the potential to support children in their learning remains untapped. Furthermore, professional competencies in terms of self-efficacy could be enhanced—for example, through mastery experiences (e.g., if preschool teachers receive support in mastering demanding situations) or verbal support (e.g., positive feedback from colleagues) (Bandura, 1977). With regard to work engagement, several job resources (e.g., job security and social support from colleagues) and personal resources (e.g., optimism) are relevant to engagement at work (Bakker, 2011). For further studies, it would be interesting to examine whether results differ when using other instruments on the level of the preschool class and focus on the preschool teacher for the assessment of the interaction quality. Furthermore, additional professional competencies (e.g., knowledge) should be considered as possible predictors of interaction quality and relationship quality. In future studies, it would also be of interest to take a closer look at several other teacher characteristics (e.g., their personality), which are likely to moderate the effects of professional competencies and explain some of the inconsistent effects.

## Data availability statement

The raw data supporting the conclusions of this article will be made available by the authors, without undue reservation.

## Ethics statement

Ethical review and approval was not required for the study on human participants in accordance with the local legislation and institutional requirements. Written informed consent to participate in this study was provided by the participants’ legal guardian/next of kin.

## Author contributions

Both authors contributed equally to the work (conception of the study, writing the first draft, data analyses, and editing the manuscript) and approved it for publication.

## Footnotes

1 National education systems use different terms for out-of-home educational settings for children up to school age, and translation into English can be challenging [e.g., see the country profiles of Austria and Sweden (Schreyer and Oberhuemer, 2017a,b)]. In Austria, the term “Kindergarten” is often used in German when referring to institutions attended by children from around 3 years of age until they start school (Hartel et al., 2019). However, the term “Kindergarten” can be misleading because in the USA it refers to facilities for children aged 5 (but not for 3 and 4 year olds) (Kamerman and Gatenio-Gabel, 2007). We therefore use the term “preschool” and the terms “preschool teachers” and “preschool children”. By “preschool,” we usually refer to institutions attended by children from about 3 years of age until school entry (Smidt, 2018).

2 With the term “professionalization model,” we refer to a notion of professionalization. There are different professionalization models: For instance, Thole and Polutta (2011) distinguish seven professionalization models, including competence-based models, evidence-based models, and reflexive models of professionalization.

3 An ethical review/approval was not required for this study.
